# Multiple myeloma remission following COVID-19: an observation in search of a mechanism (a case report)

**DOI:** 10.11604/pamj.2021.39.117.30000

**Published:** 2021-06-10

**Authors:** Daniel Antwi-Amoabeng, Mark Bilinyi Ulanja, Bryce David Beutler, Suresh Vodur Reddy

**Affiliations:** 1 Department of Internal Medicine, University of Nevada, Reno School of Medicine, Reno, Nevada, United States of America,; 2 University of Southern California, Keck School of Medicine, Los Angeles, CA, United States of America,; 3 Cancer Care Specialists, Reno, Nevada, United States of America

**Keywords:** Cancer, coronavirus, COVID-19, hematologic malignancy, case report

## Abstract

Coronavirus disease 2019 (COVID-19) represents a major challenge in the management of patients with hematologic malignancies. Individuals with plasma cell dyscrasias, including multiple myeloma, are at increased risk of developing severe disease. Furthermore, immunosuppressant agents, which represent an important component of multiple myeloma treatment, may increase the risk of serious infection; thus, treatment regimens may need to be modified in some patients. The pathogenesis of COVID-19 is incompletely understood and much remains to be established regarding cancer care in the setting of this new global health threat. We report a case of multiple myeloma remission that occurred after a single cycle of chemotherapy in a patient with COVID-19. In addition, we discuss possible mechanisms underlying this surprising observation. The findings warrant further investigation and may have important implications for the management of multiple myeloma and other plasma cell dyscrasias in the age of COVID-19.

## Introduction

The COVID-19 pandemic, caused by the novel severe acute respiratory syndrome coronavirus-2 (SARS-CoV-2), has had a major impact on the care of cancer patients. Cancer patients tend to have higher rates of COVID-19 [1] and cancer-related immunodeficiency is a known risk factor for severe disease [2]. Indeed, individuals with malignancy who develop COVID-19 frequently face prolonged hospitalization or death [3]. The underlying cause of poor outcomes among cancer patients who develop COVID-19 is thought to be two-fold: malignancy induces an aberrant immune response and chemotherapy further compromises host defense. However, although COVID-19 is often fatal in the setting of cancer, it is also conceivable that therapies administered for COVID-19 affect or enhance the response to chemotherapy. Furthermore, the intrinsic properties of SARS-CoV-2 may influence the behavior of an underlying neoplasm. The full spectrum of COVID-19 sequelae remains to be fully characterized and its effect on acute and chronic disease states-including cancer-warrants further investigation [4]. Here, we report a case of humoral and histologic resolution of multiple myeloma (MM) in a patient after a single cycle of MM therapy followed by SARS-CoV-2 infection.

## Patient and observation

**Figure 1:**
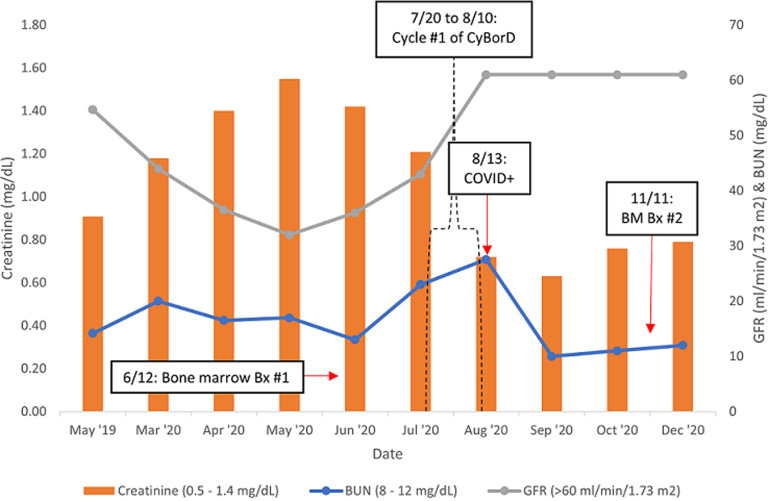
trend in renal function relative to initiation of myeloma chemotherapy and COVID-19 infection; BM= bone marrow, Bx = biopsy

**Patient information:** a 76-year-old female was referred to the oncology clinic for further evaluation of abnormal laboratory studies, including serum protein electrophoresis with M-spike elevated at 1.65 g/dL and elevated IgG-type kappa monoclonal protein on immunofixation. She was noted by her primary care physician to have progressively worsening renal function and elevated serum globulin levels over the previous year (Figure 1). She was a life-long nonsmoker, but she drank a glass of wine with dinner most nights. She denied illicit drug use. Family history was negative for hematopoietic malignancies.

**Clinical findings:** physical examination was significant only for obesity (body mass index: 41 kg/m^2^). There was no significant lymphadenopathy.

**Figure 2:**
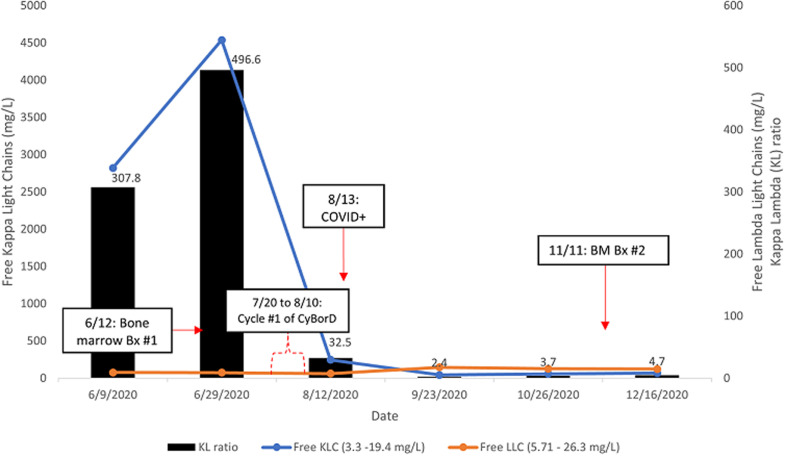
trend in serum free light chain levels and kappa-lambda free light chain (KL) ratios relative to initiation of myeloma chemotherapy and COVID-19 infection; KL ratios are shown above the bars; BM= bone marrow, Bx = biopsy

**Figure 3:**
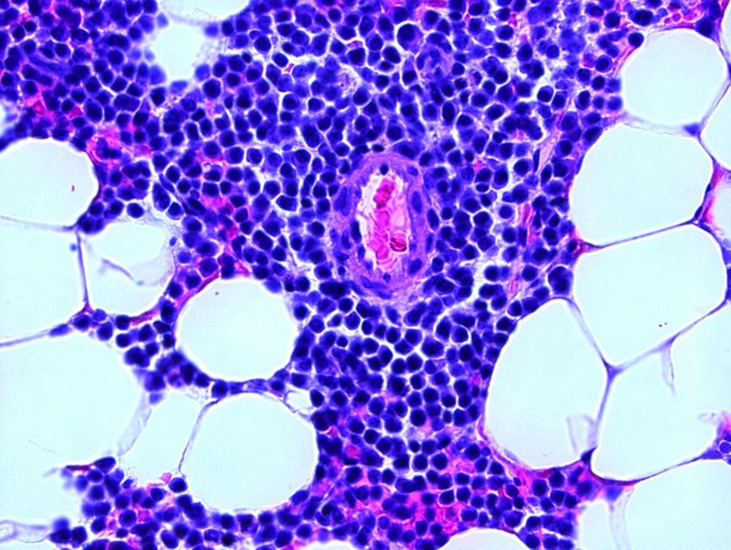
initial bone marrow biopsy at 20x magnification shows sheets of plasma cells highlighted with CD138 immunohistochemistry staining

**Figure 4:**
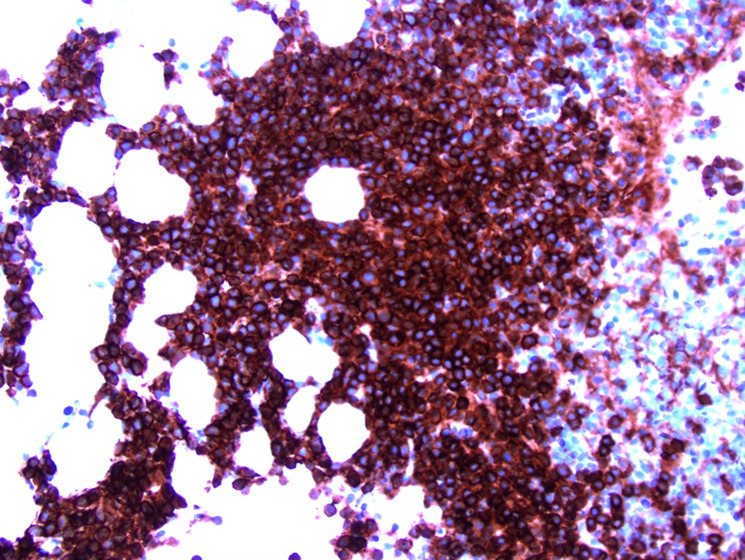
initial bone marrow biopsy at 40x magnification showing sheets of plasma cells with hematoxylin and eosin staining

**Diagnostic assessment:** a positron emission tomography scan was negative for extramedullary sites of activity or bone disease. Peripheral blood smear showed mild absolute neutropenia. There was no laboratory evidence of anemia or hypercalcemia. Total 24-hour urine protein was 1475 milligrams per day (reference range: 40 - 150 milligrams per day). Serum free light chains were markedly elevated (Figure 2). Bone marrow aspirate and core biopsy showed mildly hypercellular marrow (50%) with trilineage hematopoiesis and 80% monoclonal plasma cells; CD138 + by immunohistochemical studies; and background normal hematopoietic cells (Figure 3, Figure 4). Cytogenetic studies showed 13q and 17p deletions, conferring high risk features. The patient was diagnosed with multiple myeloma and started on a cyclophosphamide-bortezomib-dexamethasone (CyBorD) regimen due to her significant renal impairment

Three days after the last dose of cyclophosphamide, the patient presented to the emergency department with complaints of intermittent fevers, chills, sore throat, and a dry cough. Polymerase chain reaction of nasopharyngeal swab was positive for SARS-CoV-2. The patient was hospitalized overnight for observation. She had mild neutropenia (absolute neutrophil count [ANC]: 1200 K/μL [reference range: 2-7.15 K/μL], white blood cell count [WBC]: 1.9 K/μL [reference range: 4.8-10.8 K/μL]). A single dose of filgrastim 480 mcg was administered. The fevers resolved and the patient was discharged home the following day with a five-day course of levofloxacin 500 mg per os once daily and acyclovir 400 mg per os twice daily and asked to self-quarantine.

The patient returned to the emergency department seven days later, now with dyspnea and fatigue in addition to a persistent dry cough, chills, and sore throat. Vital signs were significant for fever (temperature: 101.8°F) and hypoxia (oxygen saturation: 88% on room air, requiring 4 liters of supplemental oxygen per minute to maintain oxygen saturation of 95%). Laboratory studies again showed neutropenia (ANC: 1160 K/μL, WBC: 1.6 K/μL).

**Therapeutic intervention:** she was hospitalized and started on a ten-day course of dexamethasone 6 mg per os daily as well as remdesivir (200 mg once and 100 mg daily for 4 days). In addition, a single unit of convalescent plasma was administered. She was discharged eleven days later after improvement in her respiratory status.

**Figure 5:**
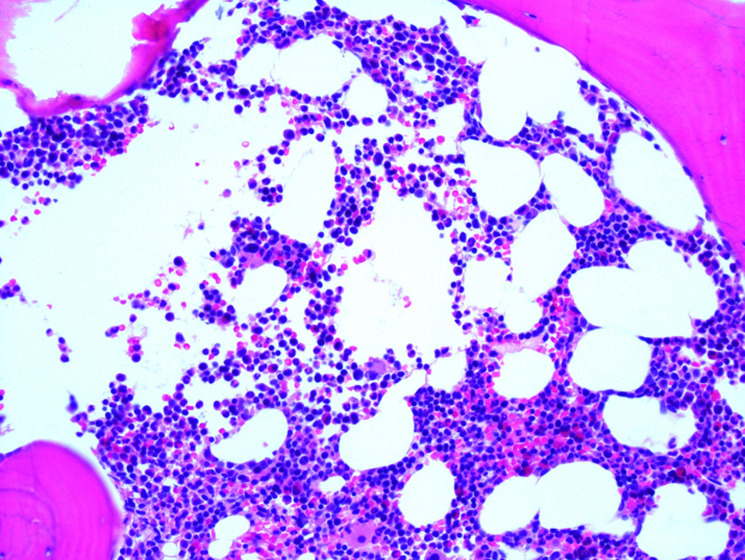
bone marrow biopsy after a single cycle of CyBorD and COVID-19 infection at 20X magnification demonstrating only scattered plasma cells on CD138 immunohistochemistry staining

**Figure 6:**
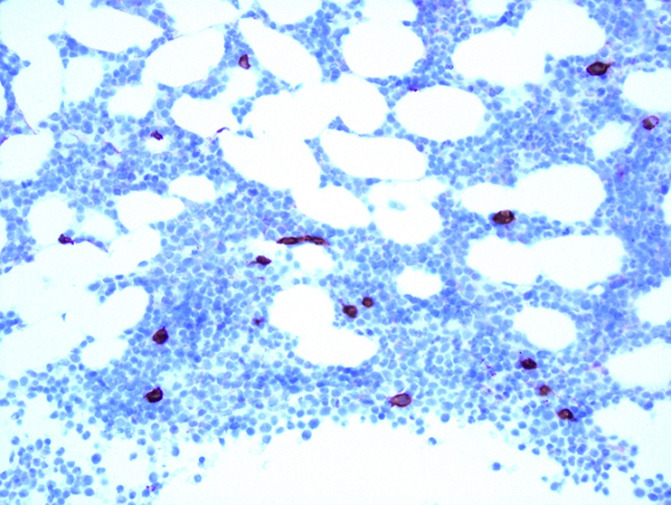
bone marrow biopsy after a single cycle of CyBorD and COVID-19 infection at 20X magnification demonstrating trilineage hematopoiesis with hematoxylin and eosin staining

**Follow-up and outcomes:** the patient was seen in the teleoncology clinic seven weeks later, where follow-up laboratory studies showed dramatic improvement in myeloma disease markers (Figure 2). She underwent another bone marrow biopsy, which showed normocellular bone marrow with the usual trilineage hematopoiesis (Figure 5, Figure 6). There was no increase in blasts or plasma cells nor significant dysplasia. Plasma cells were polyclonal by flow cytometry and fluorescence in situ hybridization. Indeed, there was no evidence of multiple myeloma. The patient continues to follow up in the oncology clinic and plans to resume chemotherapy if there is evidence of relapse. As of this writing, two months since laboratory studies showed improvement in disease markers, she remains in remission.

**Patient perspective:** the patient was delighted with the care received in the hospital and optimistic about the prospect of complete and sustained remission.

**Informed consent:** the patient was informed about the case report and willingly gave informed consent for publication of case details.

## Discussion

In this report, we describe a case of multiple myeloma (MM) remission in a patient who received a single cycle of CyBorD and subsequent developed COVID-19. The treatment response was confirmed with repeat bone marrow studies and laboratory studies demonstrating improvement in renal function and serum monoclonal protein levels. The kappa/lambda ratio began to decrease to less than 4: 1 after a single cycle of CyBorD and continued to decrease following the diagnosis of COVID-19 (Figure 2). According to the National Comprehensive Cancer Network (NCCN) guidelines and International Myeloma Working Group (IMWG) criteria, the patient met stringent criteria for complete response after just one cycle of therapy [5, 6].

The treatment response demonstrated by our patient is typically observed after four complete cycles of CyBorD therapy [7]. In a randomized phase II clinical trial by Reeder *et al*. successful treatment was defined as ≥ 40% of patients achieving a very good partial response or better after four cycles of CyBorD. The Reeder group found an 80% reduction in major protein components after two cycles (eight weeks) of CyBorD therapy. In our patient, a comparable but deep treatment response was seen after only one cycle of CyBorD and subsequent COVID-19. It is therefore conceivable that treatment of the infection and/or the infection itself altered the course of the underlying malignancy.

We postulate that one or several of the pharmacologic agents used in the COVID-19 treatment regimen-including dexamethasone, remdesivir, and convalescent plasma-may have interrupted signaling pathways needed to maintain myeloma cell production and augmented the cytotoxic effects of CyBorD, resulting in dramatic clearance of myeloma cells from the bone marrow. Secretion of IL-6 by bone marrow stromal cells is required for the growth of multiple myeloma cells [8]; this inflammatory cytokine is inhibited by dexamethasone. The dose of dexamethasone used in the CyBorD regimen is 40 milligrams daily for days 1-4, 9-12, and 17-20 on a 28-day cycle. Our patient received 6 milligrams of dexamethasone daily for ten days as part of her routine COVID-19 treatment. It is possible that these extra dexamethasone doses, which followed the first cycle of CyBorD, affected the behavior or progression of our patient´s malignancy.

An additional component of the COVID-19 treatment regimen that may have affected the behavior of MM in our patient is convalescent plasma, which is used to increase humoral immunity against structural COVID-19 proteins. MM is characterized by dysfunction in both humoral and cellular immunity, which affects immune surveillance and allows for advancement and escape of clonal cells [9]. In our patient, humoral immunity conferred by convalescent plasma may somehow have impaired MM progression.

Augmentation of MM treatment with non-chemotherapeutic agents is not without precedent. Other medications not used in traditional MM therapy have been shown to enhance clearance of myeloma cells from the bone marrow. Examples include metformin, which has been reported to decrease IL-6 expression in MM cell lines when used synergistically with dexamethasone, bortezomib, and pomalidomide [10]. In addition, tocilizumab-an anti-IL-6 monoclonal antibody-has been used in patients with COVID-19 [11], including those with MM; in one case report, authors speculated that tocilizumab may not only improve symptoms of COVID-19, but also prevent myeloma cell growth by inhibiting IL-6 signaling [12]. An alternative hypothesis to explain our patient´s dramatic improvement may be related to intrinsic properties of SARS-CoV-2. Several viruses-including parvovirus B19, hepatitis viruses, and human immunodeficiency virus (HIV)-are known to cause myelosuppression with profound cytopenia. In patients with HIV, for example, viral proteins can induce B-cell apoptosis [13]. Oncolytic viruses are also being studied. Coxsackievirus A21 (CVA21) is one such oncolytic virus that has been shown to induce lysis of MM and CD138+ plasma cells *in vitro* by exploiting intercellular adhesion molecule 1 (ICAM-1) [14]. Interestingly, SARS-CoV-2 shares many properties with CVA21: both are positive-sense single-stranded RNA viruses that are spread by aerosol transmission and exhibit multi-tissue tropism. In addition, patients with COVID-19 demonstrate increased serum ICAM-1 [15]. It therefore appears possible that the interplay between SARS-CoV-2 proteins, ICAM-1, and MM cells may have contributed to our patient´s rapid and unexpected remission.

Finally, despite the dramatic response, the above factors may not have played a major role at all. It is reasonable to suggest that our patient´s MM was very sensitive to the chemotherapy regimen and thus her remission was unrelated to COVID-19. Larger observational studies are warranted to clearly define this relationship.

## Conclusion

The effect of COVID-19 and/or COVID-19 treatment on MM remains to be definitively established. Although COVID-19 outcomes among patients with malignancy are generally poor, we describe an unusual case in which MM remission was achieved following a single cycle of CyBorD and subsequent development of COVID-19. The underlying cause of this surprising finding may be related to the COVID-19 treatment regimen-which included dexamethasone and convalescent plasma-or to intrinsic properties of SARS-CoV-2. Modification of dexamethasone dosing in patients with MM may prove beneficial. Furthermore, proteins produced by SARS-CoV-2 may have yet undiscovered oncolytic properties. Future investigation may help to clarify the relationship between COVID-19 and hematologic malignancies.
